# Bachmann bundle and adjacent pacing—Do all roads lead to Rome?

**DOI:** 10.1016/j.hrcr.2026.03.024

**Published:** 2026-04-03

**Authors:** Ankur A. Karnik

**Affiliations:** 1 HonorHealth Heart Care – Cardiac Arrhythmia Group, Scottsdale, Arizona; 2 John Shufeldt School of Medicine and Medical Engineering, Arizona State University, Phoenix, Arizona

Jean George Bachmann first described the interatrial connection that bears his name in 1916.^1^ It was further characterized by Thomas James in 1963 as a part of the anterior internodal connection system (the medial and posterior internodal pathways also connect the sinus node to the atrioventricular node).^2^ The anterior internodal tract leaves the sinus node and curves around the superior vena cava and anterior wall of the right atrium, where it becomes Bachmann bundle (BB) and subsequently bifurcates to traverse either side of the left atrial appendages. BB becomes a discrete entity at the interatrial groove.^3^

Experimental BB delay causes partial interatrial block, and BB block causes advanced interatrial block,^4^ which increases the risk for AF.^5^ Furthermore, longitudinal dissociation across BB results in reentry, which can promote supraventricular tachycardia^6^ and atrial fibrillation (AF).^7^ BB pacing has been shown to reduce progression of paroxysmal AF in a randomized study compared to right atrial appendage (RAA) pacing^8^ as well as de novo AF in a retrospective study compared to RAA and right atrial septal pacing.^9^

In this issue of *Heart Rhythm*, Brigido et al^10^ report on a patient who underwent leadless atrial pacemaker implantation at the septal base of the RAA anterior to the torus aorticus after several unsatisfactory sites along the base of the RAA and arcuate ridge were evaluated. P-wave duration during atrial pacing was 136 ms compared with 148 ms during sinus rhythm, suggesting reduced interatrial conduction delay. Pacing parameters remained excellent at 6-month follow-up. However, BB was not captured according to strict published electrocardiographic criteria: (1) similar P-wave morphology and inferior axis as sinus rhythm; (2) peaked and symmetric inferior P waves with higher amplitude than that of the sinus P wave; and (3) shorter P-wave duration compared with sinus, typically by >10 ms if the baseline interatrial conduction delay is present.^9^

Also, in this issue of *Heart Rhythm* *Case Reports*, Ogano et al^11^ purposefully implant an atrial leadless pacemaker in the anterosuperior aspect of the right atrium (RA) in 3 patients. This area originates from the primitive atrium and is surrounded by pectinated muscle and therefore safe for fixation. P-wave morphology resembled sinus morphology in all cases, but did not meet other electrocardiographic criteria for BB capture. In fact, the PR interval was prolonged compared to native conduction in one of their cases, suggesting electrical latency. Given the proximity of the anterosuperior RA to the sinus node, the authors surmised that activation proceeded through the terminal groove and subsequently through BB.

Several questions arise from these 2 thought-provoking reports. First, if BB was not directly captured, what was? As Ogano et al^11^ pointed out, the AVEIR (Abbott Laboratories, Abbott Park, IL) RA helix is 1.63 mm and unlikely to capture the epicardial fibers coalescing into BB directly. Multielectrode endocardial mapping in a canine model has shown interatrial connection not only via BB and coronary sinus but also via anterosuperior and posterinferior septal pathways.^12^ In a recent analysis of gross specimens, a consistent posterosuperior bundle was also seen, which originated from the posterior RA myocardial wall or the posterior part of the muscular sleeve of the superior vena cava. The fascicles then traversed the interatrial groove from behind the superior vena cava and joined the posterior part of BB.^13^ There is a recent report on 2 patients in whom transvenous posterosuperior bundle pacing was accomplished.^14^ It is possible that another tract or bundle was recruited by Brigido et al^10^ and Ogano et al.^11^ Second, would it be possible to capture BB if a higher output was used? This is analogous to nonselective conduction system capture at higher output as can be seen with transvenous pacing. Importantly, BB is broad and noninsulated, unlike the His-Purkinje system, and is easier to recruit. Third, given the reduction in P-wave duration with pacing, interatrial conduction was enhanced. Would this be sufficient to prevent AF? Most negative randomized trials evaluating the effect of interseptal pacing on AF occurrence (including the Septal Pacing for Atrial Fibrillation Suppression Evaluation trial) used fluoroscopic and not strict electrocardiographic criteria for BB pacing.^15^ Fourth, Brigido et al^10^ highlighted the importance of intracardiac ultrasound for placement of leadless pacemakers, especially in challenging cases, should this become standard practice? 3-dimensional mapping, which was used by our group in a case where significant RAA scar was present,^16^ may have helped in this case to identify a suitable implant site.

The Brigido et al^10^ and Ogano et al^11^ have made an important contribution to the neglected field of atrial resynchronization. These 2 studies join other publications establishing the safety and efficacy of leadless BB and adjacent bundle pacing, most recently a case series of 17 patients by Steve Bailin’s group,^17^ who previously published a seminal multicenter trial on transvenous BB pacing and AF recurrence.^8^ It appears that both direct BB and adjacent pacing can improve interatrial conduction—not all roads may lead to Rome, but many do.

## Disclosures

Dr Karnik serves as a consultant for Abbott CRM and CardioFocus.
